# Deep Learning for Strawberry Canopy Delineation and Biomass Prediction from High-Resolution Images

**DOI:** 10.34133/2022/9850486

**Published:** 2022-10-11

**Authors:** Caiwang Zheng, Amr Abd-Elrahman, Vance M. Whitaker, Cheryl Dalid

**Affiliations:** ^1^Gulf Coast Research and Education Center, University of Florida, Wimauma, FL 33598, USA; ^2^School of Forest, Fisheries, and Geomatics Sciences, University of Florida, Gainesville, FL 32603, USA; ^3^Horticultural Sciences Department, University of Florida, Gainesville, FL 32603, USA

## Abstract

Modeling plant canopy biophysical parameters at the individual plant level remains a major challenge. This study presents a workflow for automatic strawberry canopy delineation and biomass prediction from high-resolution images using deep neural networks. High-resolution (5 mm) RGB orthoimages, near-infrared (NIR) orthoimages, and Digital Surface Models (DSM), which were generated by Structure from Motion (SfM), were utilized in this study. Mask R-CNN was applied to the orthoimages of two band combinations (RGB and RGB-NIR) to identify and delineate strawberry plant canopies. The average detection precision rate and recall rate were 97.28% and 99.71% for RGB images and 99.13% and 99.54% for RGB-NIR images, and the mean intersection over union (*mIoU*) rates for instance segmentation were 98.32% and 98.45% for RGB and RGB-NIR images, respectively. Based on the center of the canopy mask, we imported the cropped RGB, NIR, DSM, and mask images of individual plants to vanilla deep regression models to model canopy leaf area and dry biomass. Two networks (VGG-16 and ResNet-50) were used as the backbone architecture for feature map extraction. The *R*^2^ values of dry biomass models were about 0.76 and 0.79 for the VGG-16 and ResNet-50 networks, respectively. Similarly, the *R*^2^ values of leaf area were 0.82 and 0.84, respectively. The RMSE values were approximately 8.31 and 8.73 g for dry biomass analyzed using the VGG-16 and ResNet-50 networks, respectively. Leaf area RMSE was 0.05 m^2^ for both networks. This work demonstrates the feasibility of deep learning networks in individual strawberry plant extraction and biomass estimation.

## 1. Introduction

Plant phenotyping is an emerging science of characterizing and quantifying the physical, physiological, and biochemical traits of crops [1]. It can provide quantitative information for assessing crop performance under specific environmental and management conditions. The most commonly used phenotyping traits include geometric parameters (e.g., plant height, crop canopy area, and leaf area index (LAI)), abiotic/biotic resistance (e.g., canopy temperature, stomatal conductance, and leaf water potential), biophysical/chemical parameters (e.g., biomass, chlorophyll, and photosynthesis), and yield [2]. The breeding process, which involves identifying the genotypes with the most desirable traits (e.g., diseases resistant and high yield), relies on the extraction and description of these traits [3, 4]. Traditional phenotypic analysis methods are labor-intensive as it requires professionals of this field to score plant samples, record plant characteristics (e.g., plant height), and manually harvest selected plants to the laboratory for further testing [5]. Over the last ten years, computer vision and robotics technology augmented with high-throughput plant phenotyping systems (HTTPs) have been developed to relieve the phenotyping bottleneck [6].

The use of multiple remote sensing platforms (satellites, drones, and ground devices) and sensors (RGB, multi- and hyper-spectral, thermal, and LiDAR) has facilitated the collection of large amounts of image data. The emergence of big data coupled with the use of machine/deep learning technique enables a rapid, non-invasive, and detailed acquisition of plant phenotyping traits throughout the whole life cycle of the crop [7]. The identification and localization of crops, fruits, and weeds from images are critical step in the subsequent extraction of plant phenotype information. The typical pipeline of machine learning algorithms is extract features from multiple color spaces then implement threshold segmentation, edge detection, and morphological processing to separate objects from background [8]. This method, although effective, often lacks robustness since it heavily depends on the quality of handcrafted features and hyperparameter tuning, especially when faced with complex environmental conditions such as lighting, weather, multi-target adhesion, and uncontrolled external interventions [9].

In recent years, deep learning, especially Convolution Neural Networks (CNN), has proven to be a powerful tool for detecting and delineating plants from remote sensing images. Mask R-CNN is one of the most popular instance segmentation algorithms [10] and has also been shown effective in many previous agricultural studies. For example, Machefer et al. [11] used Mask R-CNN model to detect and count two low-density crops (potato and lettuce) from the UAV imagery with a spatial resolution of ~2 cm. The mean average precision (*mAP*) was 0.418 for potato plants and 0.660 for lettuces in the individual plant segmentation tasks. Wang et al. [12] built a Fruits 360 Dataset using the Labelme tool and then proposed an improved Mask R-CNN framework for the detection of various fruits such as apricot, corn, and pineapple. The overall test accuracy reached 99.66%. [13, 14] applied Mask R-CNN to generate mask images of ripe strawberry fruits and presented a visual localization method to get the strawberry picking points from these masks. The average detection precision rate was 95.78% and the picking point error was within 1.2 mm, which is a valuable advancement for fruit picking robot.

Biomass is an important plant attribute that offers insights into the ability of the plant to utilize sunlight, water, and mineral nutrients to grow various tissues and organs [15, 16]. At present, the estimation of biomass through various sensing technologies has become a research hotspot in precision agriculture [17–19]. According to our literature query, there are still few studies using deep learning regression models for crop biomass prediction. Ma et al. [20] predicted the above ground biomass of winter wheat at early growth stages using pre-designed deep convolutional neural network (DCNN) with an accuracy (*R*^2^) of 0.80. Castro et al. [21] evaluated two CNN-based architectures (AlexNet and ResNet-18) in the estimation of forage biomass from high-resolution UAV RGB images and found that the prediction accuracy (*R*^2^) using the AlexNet framework can reach 0.88. Most previous studies focused on establishing models to determine the underlying relationships between various image-derived features and biomass. Commonly used image-based parameters include plant geometric indicators (canopy area, volume, plant height, shape, etc.) and spectral characteristics (individual band values, spectral derivatives, and vegetation indices), which have been proven quite effective in biomass estimation.

For example, Chen et al. [22] extracted 36 high-quality features from RGB, NIR, and fluorescence images, including geometric descriptors and physiological indicators (spectral band values and fluorescence, and near-infrared (NIR) related). Based on these parameters, the authors developed biomass prediction models for barley (*Hordeum vulgare*) using four machine learning methods: multivariate linear regression (MLR), multivariate adaptive regression splines (MARS), random forest (RF), and support vector regression (SVR). The results showed that RF performs better than other methods with prediction accuracy (*R*^2^) higher than 0.9. Quirós et al. [23] selected 6 image features derived from high-resolution multispectral imagery taken by drones as input variables for field pea (Pisum sativum L.) biomass prediction, including the Green Red Vegetation Index (GRVI), Normalized Difference Red Edge Index (NDRE), NDVI, plot volume, canopy height, and coverage. The Lasso method was implemented for prediction and the average accuracy (*R*) was approximately 0.84 [24]. Johansen et al. [25] presented a novel approach for tomato (*Solanum pimpinellifolium*) fresh shoot mass prediction. The author designed an object-based segmentation rule in eCognition software to delineate individual tomato plants and extracted several image-related variables, such as shape descriptors, vegetation indices, and entropy texture. The random forest method was adopted for biomass prediction resulting in 87.95% explained variance.

Strawberry (*Fragaria ×ananassa*) is widely appreciated all over the world in virtue of its delicious taste, high nutrition, and pleasant flavor. Several studies have been implemented to predict strawberry biomass, showing encouraging results. Guan et al. [26] applied the Structure from Motion (SfM) and Object-Based Image Analysis (OBIA) method to obtain strawberry canopy geometric indicators from high-resolution images (~5 mm), such as planimetric canopy area, canopy average height, and canopy smoothness metric. Multiple linear regression (MLR) was used for strawberry leaf area and dry biomass prediction. It showed that the prediction accuracy (*R*^2^) was about 0.79 and 0.84 for biomass and leaf area, respectively. Abd-Elrahman et al. [27] further developed geospatial analysis workflows for automated extraction of strawberry canopy size metric parameters (area, volume, average height, and height standard deviation) from high spatial resolution images and Digital Surface Model (DSM). Although this approach improved the throughput to a certain extent, it still involved sophisticated and relatively time-consuming geospatial analysis procedures and relied on models built using proprietary GIS software (ArcMap) [28]. Moreover, current attempts to estimate plant biomass using traditional machine learning and statistical methods require feature extraction and selection, involving significant manual intervention.

This study aims to establish an automatic workflow for strawberry canopy delineation and biomass prediction using deep convolutional neural networks, applied on ultra-high-resolution visible, near-infrared (VNIR) and Digital Surface Model (DSM) images. Mask R-CNN was used to identify, localize, and delineate each strawberry plant in the imagery. Canopy boundaries produced by the Mask R-CNN analysis and image data (VNIR and DSM) were input to a vanilla deep regression model (referred as to convolutional neural network with a linear regression layer) to predict biomass and leaf area. Two networks (VGG-16 and ResNet-50) were used as the base architecture for deep regression. This work automated the canopy delineation and biomass prediction processes using the powerful capabilities of deep learning, eliminating the need for feature selection and extraction.

## 2. Data Acquisition and Preparation

### 2.1. Study Site

**Figure 1:**
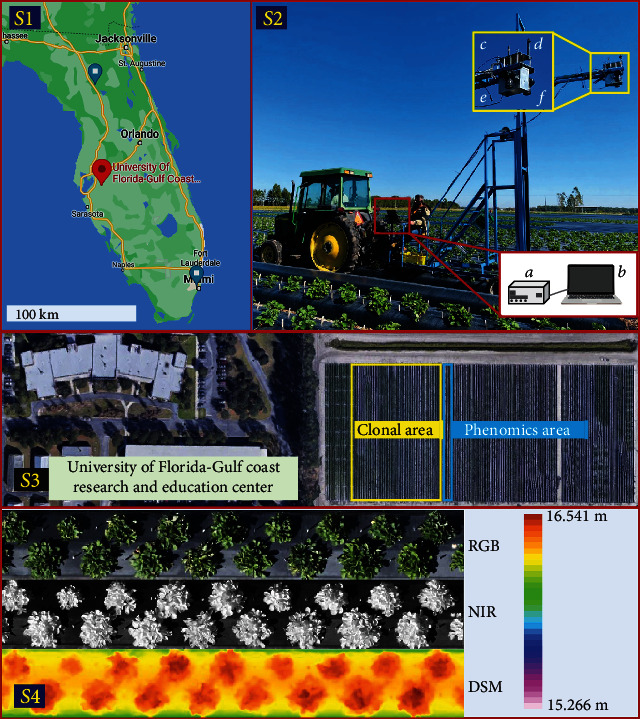
Study area and experimental setup. (S1) location of strawberry field at the Gulf Coast Research and Education Center in Wimauma, FL. (S2) Imaging platform pulled by a tractor carrying: (a) camera trigger box hardware; (b) computer; (c) timing GPS; (d) GNSS navigation system; (e) Nikon D300 RGB camera; and (f) Nikon D300 NIR camera. (S3) Strawberry farm containing two experimental areas (the clonal experiment: ~12 beds and the phenomics experiment: two beds). (S4) example of RGB, NIR orthoimages and DSM products generated from the SfM analysis.

We conducted our research on a strawberry experimental farm at the Gulf Coast Research and Education Center (GCREC) of the University of Florida in Wimauma, Florida, located at 27°45′40^″^ N and 82°13′40^″^ W. The study site included two main experiments: phenomics and clonal. The phenomics experiment area was specifically designed to test the use of high-resolution imagery in biomass estimation. It contained two adjacent 100-meter beds planted with strawberries according to commercial standards. Seventeen strawberry genotypes, representing the range of plant canopy structures in the University of Florida's strawberry breeding population, were cultivated in 34 plots. Each of the two beds consisted of 17 plots and each plot had 17 plants corresponding to the 17 strawberry genotypes. A total of 16 image acquisition sessions were conducted during the 2017/2018 and 2018/2019 Florida winter strawberry growing seasons from mid-November to the end of February of the next year. The images were acquired approximately once per week and the plants of two randomly selected plots were removed to measure leaf area and dry biomass in the lab following image acquisition. The clonal experiment comprised about 12 beds containing more than 2000 strawberry plants for the purpose of measuring various yield and quality traits in the breeding program. Three sets of images were collected for this area, captured in December, January, and February, respectively. No in-situ measurements of leaf area and dry biomass were performed on the clonal experiment plants. In addition, to verify the stability of our model, we also collected three sets of images covering both clonal and phenomics area in the 2020-2021 season. The study area and experimental setup is shown as Figure 1. The details of the data collection are shown in Table 1.

### 2.2. Image Acquisition

A ground-based imaging system presented by Abd-Elrahman et al. [29, 30] was adopted to collect high-resolution RGB and NIR images. It consists of two cameras to capture RGB and NIR images, respectively. The two cameras were deployed about 3.5 m above the ground, placed on the platform approximately 20 cm apart, and simultaneously triggered to take images every 2 s. This imaging system was propelled by a tractor along the strawberry breeding beds with a speed of 0.5 m/s. The collected images were approximately 70% side overlap, 60% forward overlap, and 0.5-millimeter raw spatial resolution. The exposure location for each image was determined by interpolating trajectories obtained by a Topcon HiperLite plus survey-grade Global Navigation Satellite System (GNSS) receiver on the platform.

Several ground control points (GCPs) were evenly distributed in the strawberry field as discussed by Guan et al. [26]. These GCPs were georeferenced with centimeter-level accuracy using a survey-grade total station and static GNSS observations. The GCPs information and thousands of raw photos were imported to the Agisoft Metashape software to generate 5 mm ground sample distance (GSD) RGB/NIR orthomosaic images and 5 mm DSM product through the Structure from Motion (SfM) analysis [31, 32]. The orthomosaic images can be used to obtain plant canopy planimetric information (e.g., length, width, area) as it has been corrected for the geometric distortion caused by topographic relief and camera tilt. The DSM product provides three-dimensional (3D) information and can be used to calculate the canopy height and volume.

### 2.3. Canopy Leaf Area and Dry Biomass Measurements

A destructive method was used to collect ground-truth (in-situ) data on total leaf area and biomass of strawberry plants within a few hours of image acquisition. Approximately every week, one plot was randomly selected from each of the two beds and plants on these two plots were harvested to the laboratory to measure total leaf area, fresh biomass, and dry biomass weight. The total leaf area of an individual strawberry plant was measured using an LI-3100 C Area Meter by summing areas of all leaves. The dry biomass of the plants was obtained after placing the plants in an oven at 65°C for 5 days. These manual measured data were considered dependent variables in the deep regression models while the images of each plant were imported as an independent variable.

## 3. Methodology

### 3.1. Experiment Workflow

**Figure 2:**
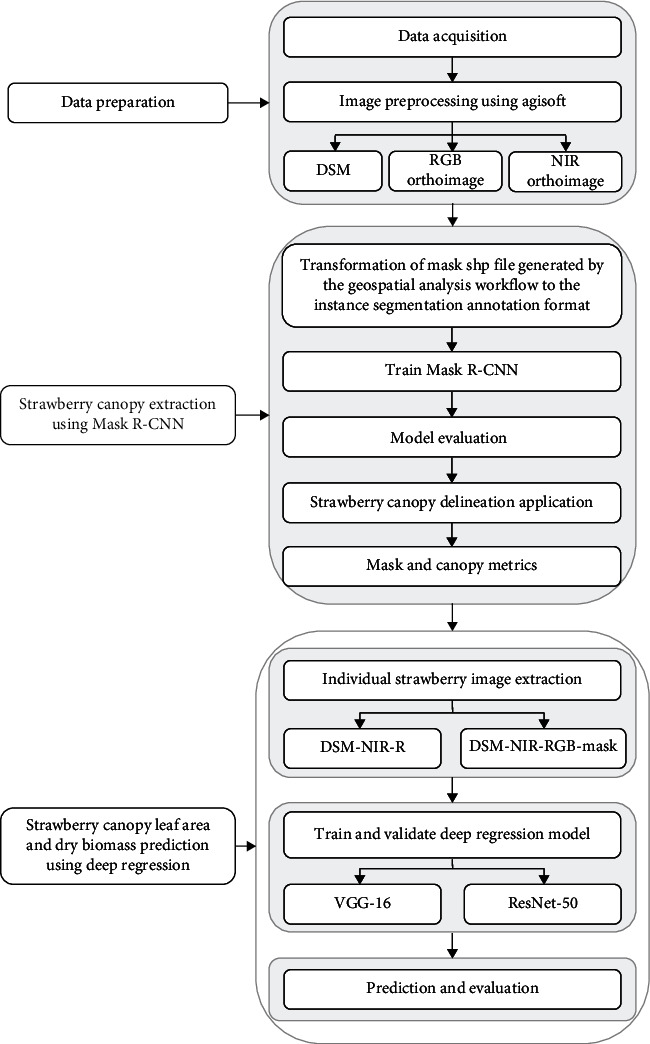
Workflow of automatic strawberry canopy delineation, and leaf area and biomass prediction from high-resolution imagery.

The experimental workflow in this study is presented in Figure 2. In the first step, the acquired images and the GCPs information were imported to Agisoft Metashape software to generate RGB, NIR orthomosaic images and DSM products through the SfM analysis. Mask R-CNN was then implemented to delineate the canopy mask for each individual strawberry plant from the orthoimages of two band combinations, including the RGB and RGB-NIR. Finally, using the images of each plant as input, deep regression was carried out to obtain canopy geometric variables (area, average height, standard deviation of height, and volume), dry biomass weight, and total leaf area. We used two band combinations of the input image (DSM-NIR-R and DSM-NIR-RGB-Mask) and two architectures of the prediction model (VGG-16 and ResNet-50) for comparison.

### 3.2. Mask R-CNN

**Figure 3:**
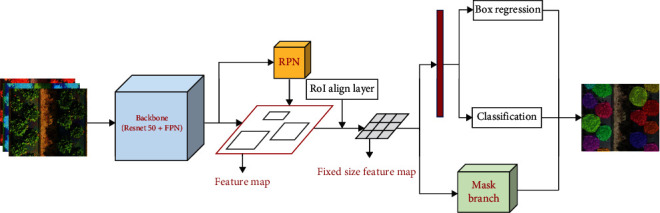
The structure of Mask R-CNN framework.

Mask R-CNN is an extension of Faster R-CNN that adds an additional branch at the end of model, which applies fully convolutional network (FCN) on the regions of interest (ROIs) to generate the target mask, thereby achieving the instance segmentation. The Mask R-CNN framework operated in three stages (Figure 3). First, the input image was imported into the feature extraction convolutional network to obtain a feature map; second, the Region Proposal Network (RPN) was applied on the feature map to generate the region proposals or candidates of interest (ROIs); finally, for each region proposal, the feature maps are performed via ROI pooling to fix size according to the region and subsequently go through the fully connected layers (FC) and FCN to realize the object detection and instance segmentation, respectively. The outputs contain the classification scores, bounding box, and binary mask.

#### 3.2.1. Backbone Structure (ResNet-50) and FPN

The input image first passed through a convolutional neural network (CNN) to generate a feature map. This CNN model was also referred to as a backbone structure, and it was designed using multiple weight layers and selected based on the trade-offs between the training speed, prediction accuracy, and computational power limitations. Since the residual network can effectively solve the gradient disappearance and improve the convergence performance when increasing the model depth, ResNet has been widely applied in the field of image processing and pattern recognition. Therefore, ResNet-50 was selected as the backbone network for strawberry canopy feature extraction in this experiment.

The advantage of CNN is that it can extract increasingly complex visual features through a hierarchical structure [8]. The underlying network in the CNN model can extract detailed image-related features (for example, edges and angles), which facilitates object detection with higher spatial dimension. The deep network can provide higher semantic information that helps determine object categories, but the spatial dimension is lower. In order to achieve multiscale feature fusion, the Feature Pyramid Network (FPN) was proposed to expand the backbone network, which is effective in the identification of objects of different sizes. Through the bottom-up, top-down, and lateral connection network, the features of each level were merged resulting in strong semantic and spatial information at the same time [33]. The top-level features of FPN were up-sampled and then fused with the bottom-level features. Each layer provided an independent convolution feature map.

#### 3.2.2. RPN and ROIs Align

The Regional Proposal Network (RPN) is a fully convolutional network presented by Ren et al. [34] to generate a set of high-quality rectangular proposals that may contain objects of interest. The features exported from the backbone network were used as input in the RPN to produce anchor boxes with object bounds and objectness scores. For each pixel, nine anchors of various area scales and length-width ratios were applied on the feature maps to obtain the regions of interest (ROIs). Then, the ROI Align was performed to extract a local feature map for each ROI, which replaced the ROI Pooling used in Faster R-CNN. It applied the bilinear interpolation to calculate the exact position of sampling point which retains the decimals. Afterwards, the maximum or average pooling was employed to unify the ROI dimensions according to the input requirements of FC and FCN. The advantage of the ROI Align approach is that it eliminated the misalignment errors caused by quantization operations in the ROI Pooling. Finally, the corresponding feature of each ROI in the feature map was subsequently extracted and imported to three prediction branches (also referred as the head layer): the FC layer for target classification, the regression layer for bounding box coordinate corrections, and the fully convolutional network (FCN) for generating object mask.

#### 3.2.3. Model Training, Loss Functions, and Hyperparameter Configurations

In this study, the strawberry plant instance segmentation was performed on the Mask R-CNN framework by Matterport [35], which was built under the TensorFlow and Keras environment. Compared with ResNet-101, ResNet-50 reduces the memory requirement and speeds up training. Therefore, it was selected as backbone network for feature extraction. To train the Mask R-CNN model, we need to generate strawberry polygon label images. A canopy delineation boundary vector file (shape file format) obtained through the geospatial analysis workflow introduced by Abd-Elrahman et al. [27] was manually edited to provide accurate strawberry canopy boundaries, and transformed to binary mask images with 0 for background and 255 for strawberry plants. Then, we converted training samples (ground-based images and labeled mask images) into the COCO annotation format datasets. The 2017-2019 image datasets were used for model training and validation and the data collected in 2020-2021 were used only for model testing. We prepared 1032 images for training (80%) and validation (20%), and 759 images for testing, summarized in Table 1. Each image was clipped from the whole orthoimage as 512 × 512-pixel tile. Meanwhile, two band combinations were compared: (1) Red, Green, and Blue (RGB) and (2) Red, Green, Blue, and near infrared (RGB-NIR). For each band combination, the number of image and instance samples is summarized in Table 2.

The total loss of the training process is divided into two parts: (1) the loss in the RPN network, which consists of anchors classification loss and bounding box regression loss; (2) the loss in the mask branch, which includes the mask loss, class loss, and bounding box regression loss. The hyperparameter configuration used in the study is (1) weights for the above five losses are all set to 1; (2) Adam optimizer is set with a learning rate of 0.001, momentum of 0.9, and weight decay of 0.0001; (3) 100 number of epochs; (4) 256 ROIs per image; (5) 500 and 100 steps per epoch during training and validation process, respectively; and (6) five anchor box scales of 16, 32, 64, 128, 256. The marker-controlled watershed algorithm introduced by Abd-Elrahman et al. [27] was implemented in the ArcMap platform and used as a baseline method for comparison with the Mask R-CNN model.

### 3.3. Deep Regression

Regression analysis is a technique for predictive modeling of continuous values by establishing the relationship between the dependent variable and the independent variable in the dataset [36]. For predictive tasks in the computer vision field, a traditional idea is to extract various features from the image and then perform regression analysis to make predictions. In the past decade, deep learning networks have been progressively developed and applied in the image recognition field for uses including classification and object detection. Researchers have also tried to take the advantage of the strong image feature expression abilities of deep learning architectures for continuous predictions. For example, Lathuilière et al. [37] replaced the last softmax layer of VGG-16 and ResNet-50 with a fully connected regression layer using linear or sigmoid activations and named this type of architecture as vanilla deep regression. It was applied on four datasets (Biwi, FLD, Parse, and MPII) for facial landmark detection and head pose estimation. The author used different data preprocessing strategies and experimented with different numbers of fine-tuned layers. The results demonstrated high performance of the deep regression (referred as to convolutional neural network with a linear regression layer) in predicting single variables. Huang et al. [38] employed the deep learning model to estimate the population intensity from satellite images and discussed the impact of model and neighborhood selection on the prediction performance.

#### 3.3.1. Model Structure

**Figure 4:**
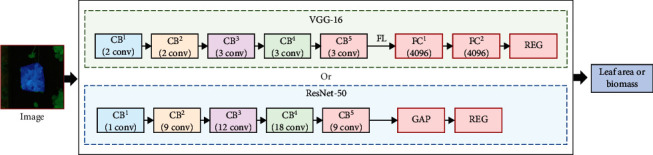
The pipeline of strawberry leaf area and biomass prediction using the deep regression model. *CB*^*i*^ represents the *i*^th^ convolutional block. *FC*^*i*^ represents the *i*^th^ fully connected layer with a dropout rate of 50%. *FL* represents a flatten layer which transforms a 2D feature map into a vector. *GAP* represents a global average pooling layer. *REG* is the regression layer.

This study aims to import individual plant images extracted by the Mask R-CNN to the vanilla deep regression model for strawberry canopy leaf area and dry biomass prediction. Two architectures were used as the backbone structures: VGG-16 and ResNet-50, which is shown in Figure 4.

#### 3.3.2. Data Preparation

Using the Mask R-CNN model discussed in Section 3.2, each strawberry plant was assigned a unique mask segmentation. According to trade-off between the spatial resolution of imagery (5 mm) and the plant size, we cropped a 128 × 128 window for each plant based on the center of the canopy mask and then resized the images to 224 × 224 pixels, which fits to the input dimension of VGG-16 and ResNet-50 model. The research conducted by Abd-Elrahman et al. [27] and Guan et al. [26] has proved that the strawberry biomass is highly correlated to the canopy geometric information, such as canopy area and plant height. Therefore, the DSM product, canopy mask, RGB image, and the NIR band were combined as the input dataset, which contained the spatial and spectral information simultaneously. Since the ImageNet pre-trained weights for VGG-16 and ResNet-50 model required an input dimension of 224 × 224 × 3 pixels, the DSM-R-NIR band combination was used as the input. We also imported the image of six bands (DSM, RGB, NIR, and Mask) to the model without the ImageNet pre-trained weights to compare the result.

**Figure 5:**
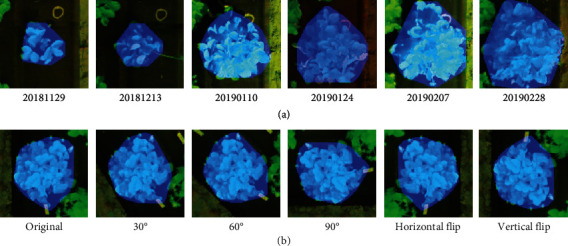
An example of the input images (R-NIR-Mask): (a) strawberry canopy samples of different growth stages; (b) a preprocessing example of input images, and it was rotated 30°, 60°, and 90° and mirrored horizontally and vertically.

The field data collected during the 2017-2018 and 2018-2019 seasons were used for the model training and validation in the study, which included approximately 1,000 in-situ biomass samples from the phenomics experiment. Since the deep learning architectures contain a huge number of parameters, these samples were not enough for training. Therefore, we adopted two ways to increase the sample size (Table 3): (1) The samples in the clonal experiment were prepared for model pre-training. The geospatial analysis workflow introduced by Abd-Elrahman et al. [27] was applied to extract canopy size metrics parameters for the plants in the phenomics and clonal experiment, including area, volume, average height, and height standard deviation. Using the phenomics experimental dataset, a multiple linear regression model (MLR) with an accuracy higher than 70% was built, which was used to predict the leaf area and dry biomass values of plants in the clonal area. (2) The rotation and mirroring operations were employed for phenomics training data augmentation. We considered all possible image rotations in the interval of [0, 90°] with a step of 30° in addition to the horizontal and vertical flip as shown in Figure 5(b). Besides, we evaluated the robustness and stability of the deep regression model using three sets of image data acquired during the 2020-2021 growing season, which contains about 100 plant samples.

#### 3.3.3. Model Training

We set the hyperparameter of the model and the configuration of other network variants according to the suggestions of Lathuilière et al. [37] and our experimental results. The Adam optimizer with a learning rate of 0.00025 was used to train the model of both VGG-16 and ResNet-50 architectures. Due to the GPU memory limitations, we chose a batch size of 128 and 32 for VGG-16 and ResNet-50, respectively. A batch normalization was added before the activation of *FC*^2^ in VGG-16 and a more recent layer normalization (LN) was employed in ResNet-50. As for dropout (DO), it was not used in ResNet-50, but was added in the *FC*^1^ of VGG-16. Finally, the Mean Squared Error (MSE) was adopted as the loss function at training time.

### 3.4. Accuracy Analysis

To evaluate the Mask R-CNN performance, various accuracy metrics were implemented in the test experiment. The precision (*P*) and recall rate (*R*) were used for the evaluation of object detection performance, which are shown below:

(1) $$\begin{array}{c} \begin{array}{c} \text{Precision}=\frac{TP}{TP+FP},\\ \text{Recall}=\frac{TP}{TP+FN}, \end{array} \end{array}$$

where the true positive (*TP*) is the number of cases that are positive and identified as positive, the false positive (*FP*) is the number of cases that are negative but identified as positive, and the false negative (*FN*) is the number of cases that are positive but identified as negative. There, the precision represents the ratio of the number of correctly identified positive instances (*TP*) to the total number of positive predictions (*TP* + *FP*). The recall rate means the ratio of the number of correctly identified positive instances to the total number of positive instances.

The mean intersection over union rate (*mIoU*) was used to evaluate the instance segmentation performance of Mask R-CNN, which is defined as follows:

(2) $$\begin{array}{c} mIoU\left(\%\right)=\frac{A_{O}}{A_{U}}, \end{array}$$

where *A*_*O*_ indicates the overlap area between the predicted and ground-truth mask, and *A*_*U*_ is the union area between the predicted segmentation mask and the ground truth.

Regarding the predictive models, the ten-fold cross-validation strategy was adopted to assess the performance of the multilinear regression model. Since it would be very time-consuming to implement cross-validation on the deep regression model, we split the whole 2017-2019 dataset into training, validation, and test using ratios of 70%, 15%, and 15%, respectively. Then, we performed rotation and mirroring on the training and validation datasets to increase the number of samples. The MLR was considered a benchmark for comparison with the deep regression approach. The coefficient of determination (*R*^2^) and Root Mean Square Error (*RMSE*) was used to assess the predictive power of the models.

## 4. Result

All our experiments were implemented under the deep learning framework of Tensorflow 2.1.0 and Keras 2.3.1, with an Nvidia TITAN X (Pascal generation) for GPU acceleration. Strawberry canopy delineation and biomass prediction experiments were carried out separately, and the results are as follows.

### 4.1. Strawberry Canopy Delineation Using Mask R-CNN

**Figure 6:**
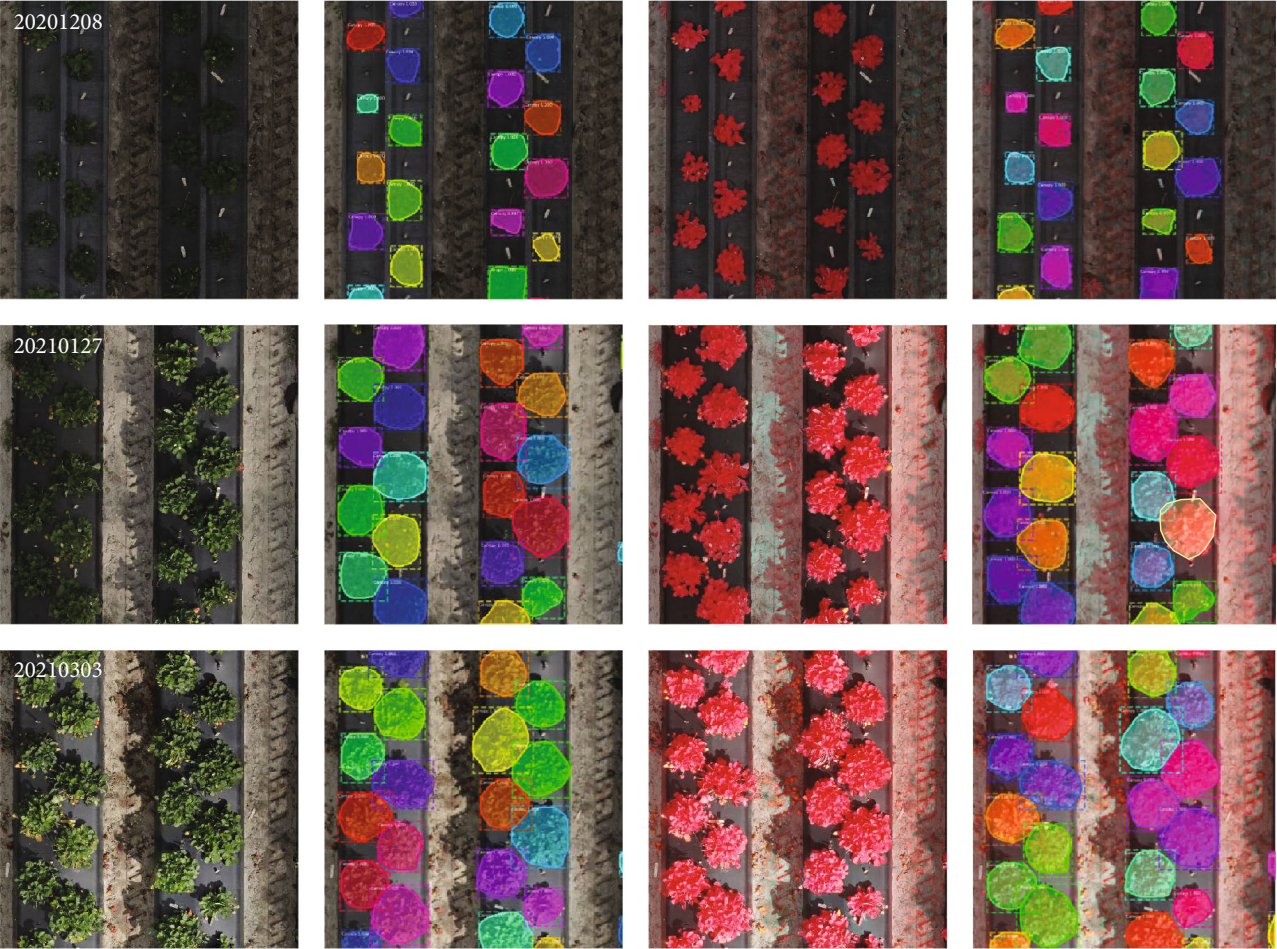
Strawberry canopy instance segmentation examples using 2020-2021 testing dataset.

The training time for Mask R-CNN was about 10 h, and the model loss converged as shown in Figure S1. From Table 4, we can see that the average precision and recall rates of Mask R-CNN model using 2020-2021 test images were 97.28% and 99.71% for RGB images and 99.13% and 99.54% for RGB-NIR images, respectively. Almost all the strawberry canopies were detected, and the illumination changes caused by differences in solar radiation during data collection seem to have a little effect on the instance segmentation performance of the model for both the RGB and RGB-NIR images (refer to Figure S2-S4). The high recall rate proves that Mask R-CNN can reliably detect all strawberry plant objects of interest. The instance segmentation results of 100 test images showed that the average *mIOU* rates reached 98.32% and 98.45% for the RGB and RGB-NIR images, respectively. An example of strawberry canopy segmentations is shown in Figure 6.

**Figure 7:**
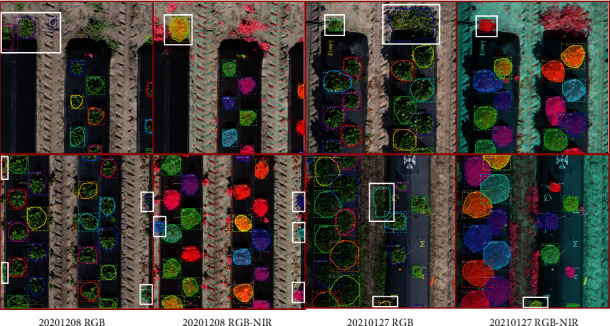
Examples of grass misidentified as strawberry canopies shown in the white box.

However, some grasses around the bed were identified as strawberry plants as shown in Figure 7. In general, the false detection of grass mainly occurred at the strawberry ripening stage and did not significantly affect the performance of Mask R-CNN on the strawberry canopy detection task. This problem could probably be solved if more of the grass-labeled images were used for model training. The above results demonstrated that Mask R-CNN performed well for strawberry detection from both RGB and RGB-NIR images. The RGB-NIR band combination seems to perform better in the grass misdetection than RGB images, which may be due to the fact that NIR can better reflect the differences between various vegetations. The marker-controlled watershed algorithm introduced by Abd-Elrahman et al. [27] reached higher precision (99.78%) and recall rates (100%) since it relied on an input vector file containing center points of all strawberry plants. However, this method has lower *mIoU* rate (92.54%). By contrast, Mask R-CNN provided more precise strawberry canopy boundaries.

### 4.2. Strawberry Canopy Metrics Calculation

Table 5 summarizes the statistical data of the image-derived canopy geometric variables, as well as the dry biomass weight and leaf area obtained through destructive measurements. The above four parameters were considered independent variables in a multilinear regression (MLR) of strawberry plant leaf area and biomass prediction. Ten-fold cross-validation was adopted to evaluate the performance of the MLR model. Multilinear regression model was used in this study as a benchmark for comparison with the deep regression architectures. We also applied the MLR model on the plants growing in the clonal area to obtain the approximate values of leaf area and biomass for pre-training deep regression models.

**Figure 8:**
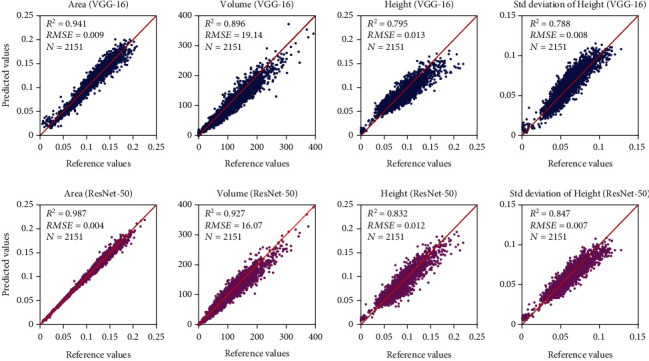
Scatter plots of reference and predicted values of strawberry geometric variables using VGG-16 and ResNet-50 architectures. The unit for canopy area, volume, averaged height, and std of plant height is square meter (m^2^), cubic centimeter (cm^3^), meter (m), and meter (m), respectively. The import was using the DSM-RGB-NIR-Mask image.

Figure 8 shows scatter plots of prediction and reference values for strawberry plant geometric variables. The prediction accuracy (*R*^2^) of canopy area, volume, height, and std deviation of height reached 0.941, 0.896, 0.795, and 0.788 using VGG-16 architecture, and 0.987, 0.927, 0.832, and 0.847 using ResNet-50 architecture, respectively. The results indicate that the deep regression model is effective in automatically extracting the geometric parameters of strawberry plants.

### 4.3. Strawberry Canopy Leaf Area and Biomass Prediction

**Figure 9:**
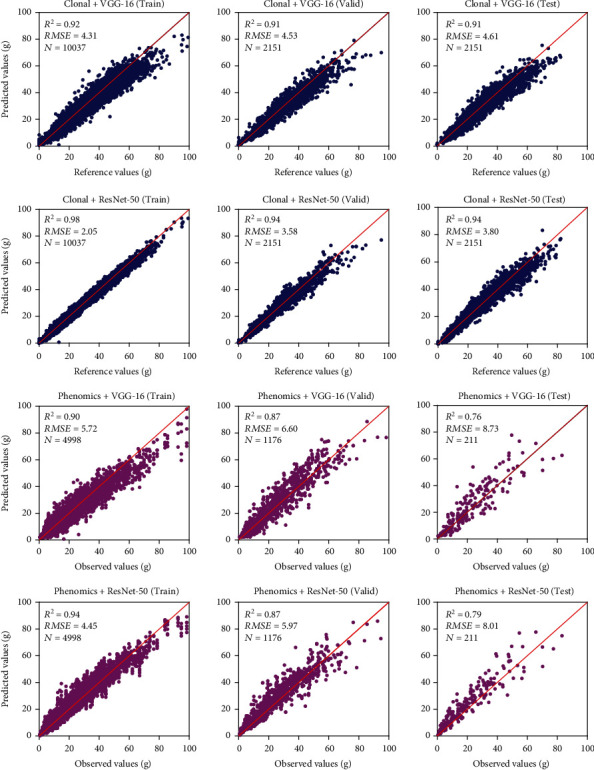
Scatter plots of reference and predicted values of strawberry dry biomass weight using VGG-16 and ResNet-50 architectures. The unit is grams (g). For the clonal area plants, the reference values were computed using multilinear regression analysis of four geometric variables (canopy area, volume, averaged height, and std of plant height). For the phenomics experiment samples, the observed values represent manually measured plant dry biomass weight. The import was using the DSM-RGB-NIR-Mask image.

**Figure 10:**
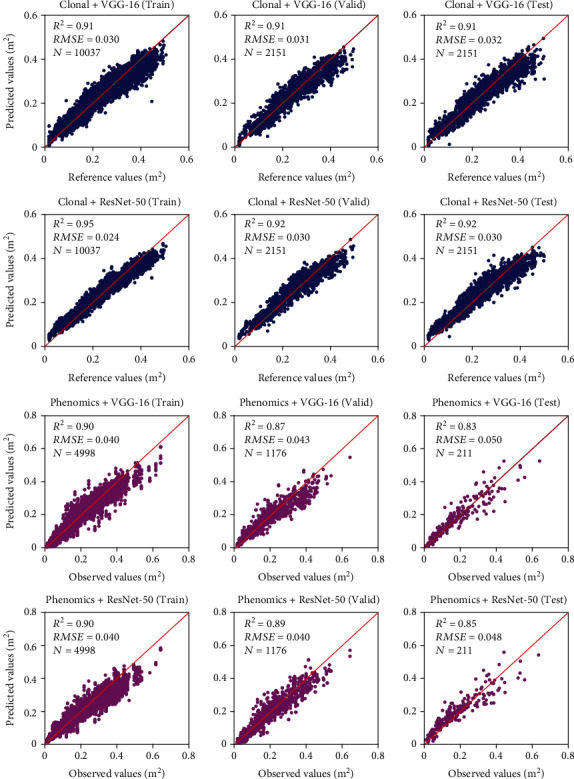
Scatter plots of reference and predicted values of strawberry canopy total leaf area weight using VGG-16 and ResNet-50 architectures. The unit is square meter (m^2^). For the clonal area plants, the reference values were computed using multilinear regression analysis of four geometric variables (canopy area, volume, averaged height, and std of plant height), while for the phenomics area samples, the observed values represent manually measured plant leaf area. The import was using the DSM-RGB-NIR-Mask image.

**Figure 11:**
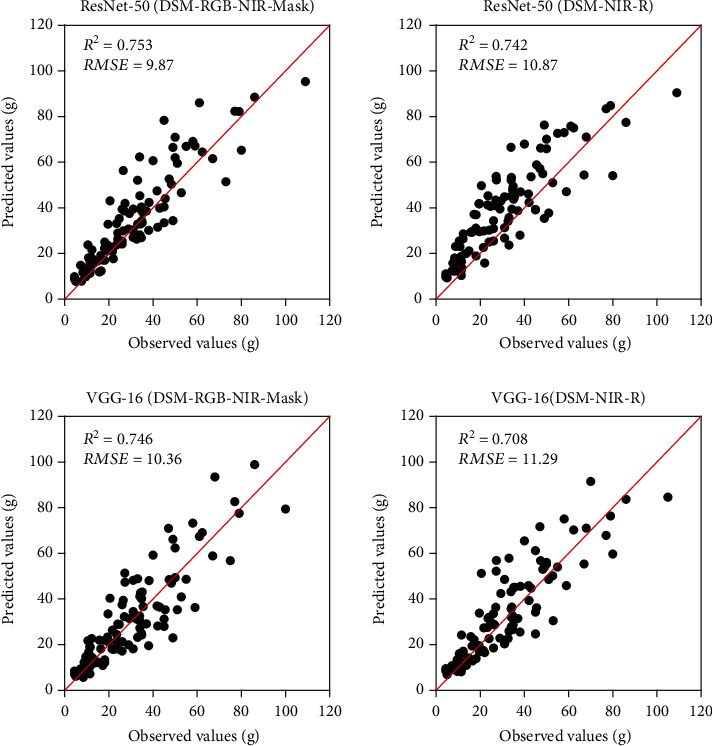
Scatter plots of reference and predicted values of strawberry canopy dry biomass weight using the 2020-2021 phenomics dataset. The unit is grams (g). Two deep regression architectures and two band combinations were compared.

Figures 9 and 10 show scatter plots between prediction and reference values for dry biomass weight and leaf area using the 2017-2019 dataset, respectively.  Table 6 compares the predictive performance of dry biomass weight and leaf area using various models and data input. For the plant samples in the clonal area, the prediction accuracy (*R*^2^) of the leaf area and dry biomass was higher than 0.9 for both VGG-16 and ResNet-50 architecture. The results indicated that deep regression models were capable of reproducing the entire process of extracting canopy geometric variables from images and modeling canopy biophysical parameters (MLR in this study). For the phenomics experiment, the *R*^2^ and *RMSE* of dry biomass in the test dataset were 0.76 and 8.73 g using VGG-16 architecture, and 0.79 and 8.31 g using ResNet-50 architecture, respectively. Similarly, the leaf area modeling yielded *R*^2^ values of 0.82 and 0.84, and RMSE of 0.05 m^2^ (for both architectures) using the VGG-16 and ResNet-50 architecture, respectively. We also used the 2020-2021 phenomics dataset to test the deep regression models, as shown in Figure 11. Overall, the previously trained model still produces acceptable prediction results with an accuracy (*R*^2^) higher than 0.75. The discrepancies compared to 2017-2019 test results may be due to the selections of new strawberry varieties in the 2020-2021 season.

## 5. Discussion

Biomass and total leaf area are important but complex biophysical traits that can be used to monitor the health and nutritional status of crops in precision agriculture. These two parameters are also key factors in breeding and genetic research to assess plant growth, yield potential, and plant regeneration ability. The primary aim of this study is to apply deep learning technologies to rapidly and automatically predict dry biomass weight and total leaf area of individual strawberry plants from images, which helps increase the throughput of phenotyping. In fact, several previous studies conducted by Guan et al. [26] and Abd-Elrahman et al. [27] have demonstrated that the geometric traits of plants extracted from high-resolution imagery are effective in the estimation of strawberry biomass and leaf area. However, their approaches contain many complicated procedures which require substantial human effort. We used Mask R-CNN and deep regression models in the task of strawberry canopy delineation and dry biomass/leaf area prediction. This is a good attempt to use deep learning models to directly and automatically predict canopy biophysical parameters of bush crops without extracting any features from the image. The results of this experiment are contributing to the rapid acquisition of strawberry biomass using deep learning methods in the breeding program, which can also be easily transferred to other plants.

In terms of canopy delineation of individual plants, the experimental scenario in this study is relatively simple as the background interference only includes bed, soil, and sometimes grass. The most difficult problems are the multi-canopy adhesion and overlapping/interference as well as the shadow caused by the illumination difference. The Mask R-CNN appears capable of overcoming the above difficulties in multiple growth stages of strawberry plants. Compared with the marker-controlled watershed algorithm introduced by Abd-Elrahman et al. [27], Mask R-CNN does not rely on human interventions, provides more accurate strawberry canopy boundaries, and exhibits strong versatility and robustness even in complex environments.

For deep regression modeling, we choose to import two types of data with different band combinations: DSM-R-NIR and DSM-RGB-NIR-Mask to compare the prediction performance. Each experiment was trained three times and an average result was taken. Meanwhile, the multilinear regression model was also performed as benchmark using ten-fold cross-validation. The result is summarized as Table 6. With respect to the network variants, we conclude that the ResNet-50 was generally more stable and worked better than VGG-16 as the behavior of VGG-16 was more variable in the training process. Of note, the ResNet-50 has a faster convergence speed than VGG-16 in a short training time. This result may be due to the design of identity shortcuts in ResNet, which augmented the network depth but reduced the number of parameters. Regarding data import, six-band images seemed to achieve a little higher prediction accuracy than three-band images. This result also demonstrates that the DSM-NIR-R images were sufficient to provide the three-dimensional spatial information of individual strawberry plants to the network. This result indicates that the limited geometric information in the image itself may be the ultimate limit to prediction accuracy. Unexplained error could be caused by differences in the canopy leaf density among genotypes that are not captured in the images. Overall, the deep regression models showed similar predictive power to the MLR method. Although the deep learning model does not improve the prediction accuracy and is complex, this method avoids the process of feature extraction and also has great potential for fully automated strawberry biophysical parameter estimation.

Remote sensing data collection and deep learning-based big data analysis methods have broad application prospects in precision agriculture and plant phenotyping. Recent advances in deep learning analysis in the field of image recognition provide more advanced frameworks for crop instance segmentation, such as the YOLO series [39] and the transformer-based methods [40]. In addition to the RGB images, high spectral dimension data have the potential to improve predictive model performance in the future. More CNN backbone networks such as Xception (Chollet et al., 2017), DenseNet (Iandola et al., 2014), and EfficientNet (Tan et el., 2019) can be evaluated and applied to the estimation of plant biophysical parameters from remote sensing images, especially for UAV images with coarse spatial resolution but more spectral information.

## 6. Conclusions

Phenotyping of field crops based on high-resolution imagery has become a hot research topic in recent years. Most of the current research has focused on extracting representative image-derived features and then importing these parameters to various regression or machine learning models to predict parameters such as plant leaf area and biomass. This manuscript presents a workflow that includes state-of-the-art deep learning methods for strawberry canopy delineation and leaf area/biomass prediction based on ground-based high-resolution imagery. Mask R-CNN was used for individual strawberry plant extraction, and a deep regression approach with two architectures (VGG-16 and ResNet-50) was adopted to directly estimate leaf area and biomass. For dry biomass prediction, *R*^2^ values were approximately 0.76 and 0.79, and the RMSE values were 8.73 and 8.31 g using VGG-16 and ResNet-50 networks, respectively. For leaf area, the *R*^2^ values were 0.82 and 0.84, and the RMSE values were both 0.05 m^2^ using VGG-16 and ResNet-50 networks, respectively. These results demonstrate the feasibility of deep regression models for predicting plant biophysical parameters without any intermediate feature (e.g., canopy height and area) extraction. Future research could apply deep learning and deep regression to the phenotyping of other traits and plant species as well as to lower-resolution images from drones.

## Figures and Tables

**Table 1:** Characteristics of the ground-based image set collected for this study.

Acquisition time	Covering area	Number of strawberry plant instances	If in-situ biomass measured?
2017-2018 season (16 weeks, once a week)	Phenomics (2 beds)	512	Yes
2017-2018 season (2 weeks, once a week)			
01/31/2018	Clonal (10 beds)	2035	No
02/27/2018	Clonal (10 beds)	2035	No
2018-2019 season (16 weeks, once a week)	Phenomics (2 beds)	512	Yes
2018-2019 season (3 weeks, once a week)			
12/10/2018	Clonal (16 beds)	3717	No
01/31/2019	Clonal (14 beds)	3278	No
02/28/2019	Clonal (14 beds)	3278	No
2020-2021 season (3 weeks, once a week)			
12/08/2020	Phenomics (2 beds)	34	Yes
	Clonal (10 beds)	3110	No
01/27/2021	Phenomics (2 beds)	34	Yes
	Clonal (10 beds)	2873	No
03/03/2021	Phenomics (2 beds)	34	Yes
	Clonal (10 beds)	2873	No

**Table 2:** Mask R-CNN training samples preparation.

	Number of images	Total number of instances	Acquisition time	Image size
Train	825	11474	2017-2019	512 × 512
Validation	207	2876		
Test				512 × 512
For precision and recall rate statistics	759	8958	12/08/2020 01/27/2021 03/03/2021	
For *mIoU* calculation	100	1392		

**Table 3:** Characteristics of data samples for deep regression.

Season	Data	Sample size		Usage
		Original	Rotated	
2017-2018	Clonal data (image collected in 01/31/2018 and 02/27/2018)	4066		Model pre-training
	Phenomics data	532	3192	Model fine tuning and validation

2018-2019	Clonal data (image collected in 12/10/2018, 01/31/2019, and 02/28/2018)	10272		Model pre-training
	Phenomics data	522	3132	Model fine tuning and validation

2020-2021	Phenomics data (image collected in 12/08/2020, 01/27/2020, and 03/03/2021)	102		For model testing only

**Table 4:** Evaluation metrics of Mask R-CNN model using the 2020-2021 dataset.

Model	Image	Precision (%)	Recall (%)	*mIoU* (%)
Mask R-CNN	RGB	97.28%	99.71%	98.32%
	RGB-NIR	99.13%	99.54%	98.45%
Marker-controlled watershed algorithm		99.78%	100%	92.54%

**Table 5:** Statistics of image-derived and in-situ total leaf area and dry biomass variables of the whole dataset.

Descriptive statistics	Image-based variables				In-situ	
	Canopy area (m^2^)	Average Heights (m)	Std deviation of height (m)	Volume (cm^3^)	Dry biomass (g)	Leaf area (cm^2^)
Min	0.04	0.001	0.004	0.80	0.5	100.07
Max	0.37	0.22	0.13	456.39	139.5	6427
Range	0.33	0.22	0.13	455.60	139	6326.93
Mean	0.13	0.06	0.06	83.78	23.21	1939.53
Median	0.13	0.06	0.06	76.19.	19.35	1749.13

**Table 6:** Predictive performance of dry biomass weight and leaf area using various models and data input applied to the Phenomics experiment dataset.

Target	Different models and import settings		Statistics	
			*R* ^2^	*RMSE* (g or m^3^)
Dry biomass	VGG-16	DSM-RGB-NIR-mask	0.752	9.155
		DSM-R-NIR	0.741	9.409
	ResNet-50	DSM-RGB-NIR-mask	0.782	8.810
		DSM-R-NIR	0.773	8.934
	MLR (ten-fold cross-validation)		0.779	8.519

Leaf area	VGG-16	DSM-RGB-NIR-mask	0.838	0.0487
		DSM-R-NIR	0.820	0.0513
	ResNet-50	DSM-RGB-NIR-mask	0.841	0.0486
		DSM-R-NIR	0.833	0.0495
	MLR (ten-fold cross-validation)		0.839	0.0495

## Data Availability

The data that support the experiments of this study are available from the Gulf Coast Research and Education Center (GCREC) of the University of Florida. Restrictions apply to the availability of these data. Data (imagery and ground-truth biomass measurements) are available from the corresponding author with the permission of GCREC.

## Supplementary Materials

Supplementary Materials Figure S1. The loss function curve for (a) RGB images and (b) RGB-NIR images Figure S2. Strawberry canopy instance segmentation examples for the image collected at 20201208 Figure S3. Strawberry canopy instance segmentation examples for the image collected at 20210127 Figure S4. Strawberry canopy instance segmentation examples for the image collected at 20210303.
