# Supplementary material for: Deep Learning for Strawberry Canopy Delineation and Biomass Prediction from High-Resolution Images
Source: Plant Phenomics. 2022 Oct 11;2022:9850486. doi: 10.34133/2022/9850486 (PMC9595049; doi:10.34133/2022/9850486)
Supplement: Supplementary Materials — Figure S1. The loss function curve for (a) RGB images and (b) RGB-NIR images Figure S2. Strawberry canopy instance segmentation examples for the image collected at 20201208 Figure S3. Strawberry canopy instance segmentation examples for the image collected at 20210127 Figure S4. Strawberry canopy instance segmentation examples for the image collected at 20210303. [file 9850486.f1.docx]

Supplementary files


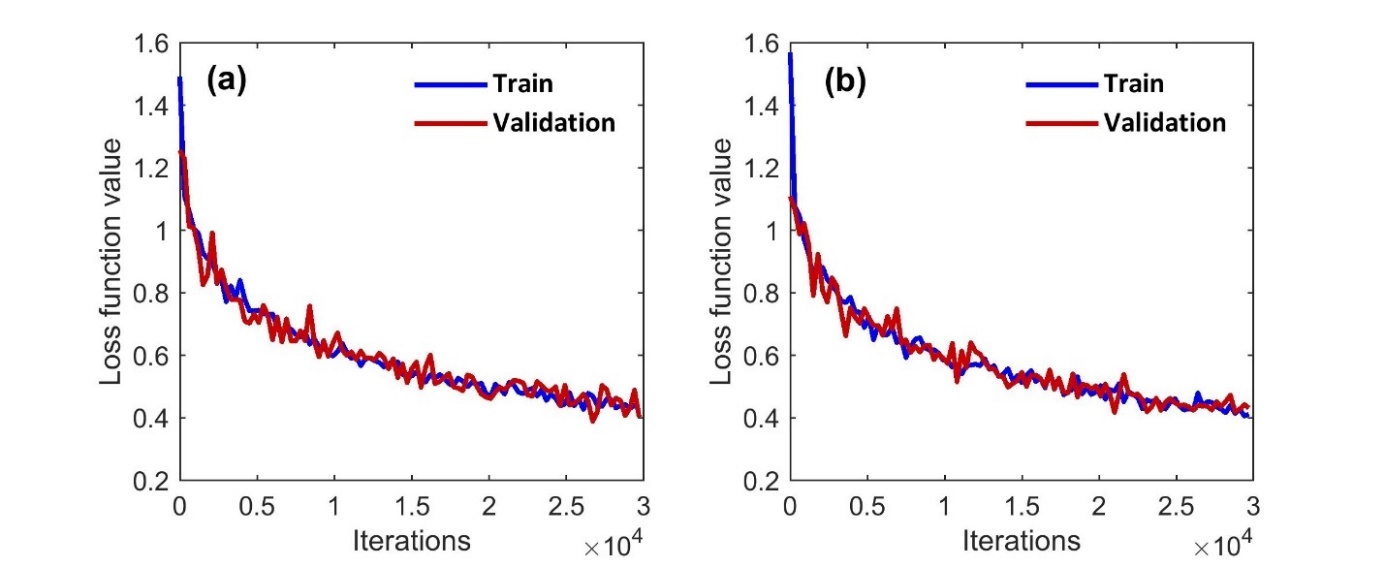


**Figure S1.** The loss function curve for (a) RGB images, and (b) NIR-R-G images


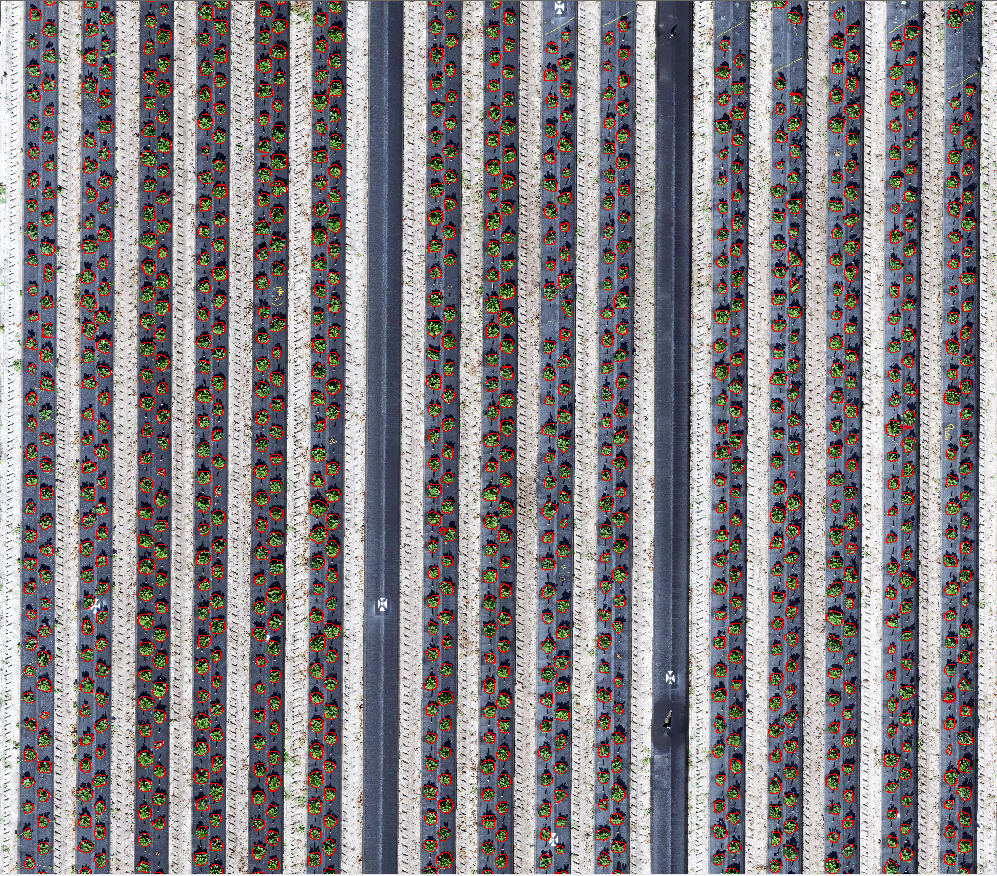


**Figure S2.** Strawberry canopy instance segmentation examples for the image collected at 20201208


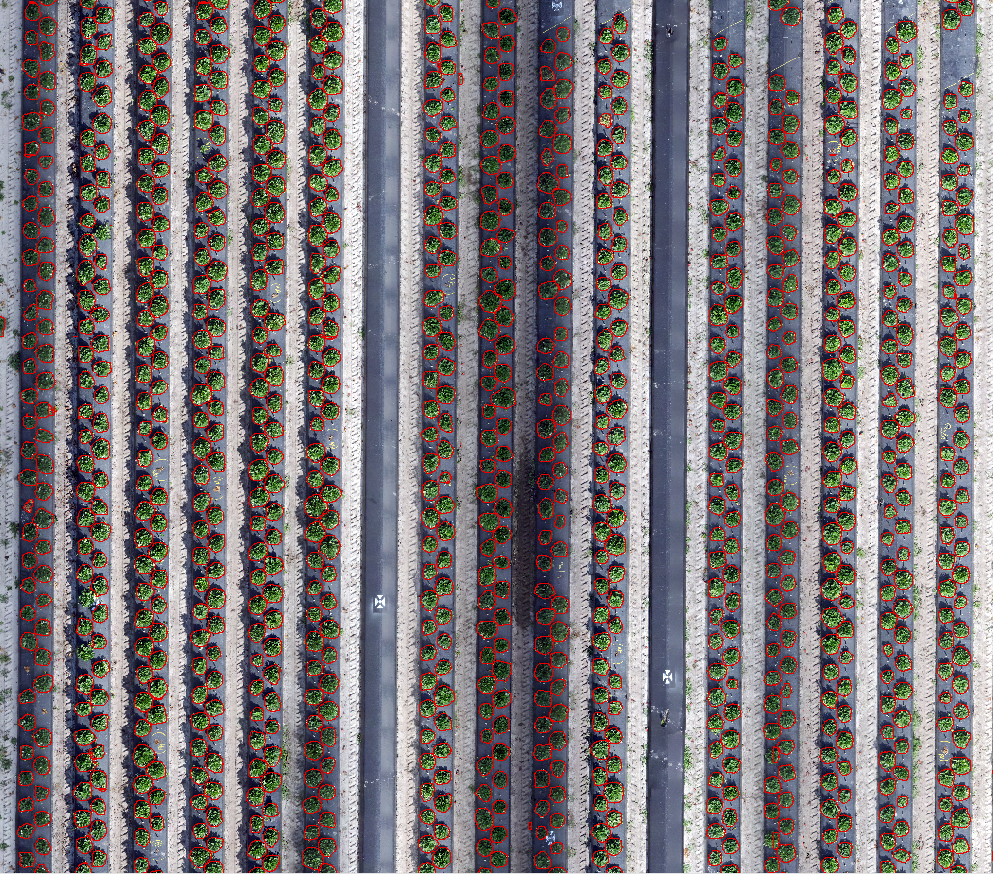


**Figure S3.** Strawberry canopy instance segmentation examples for the image collected at 20210127


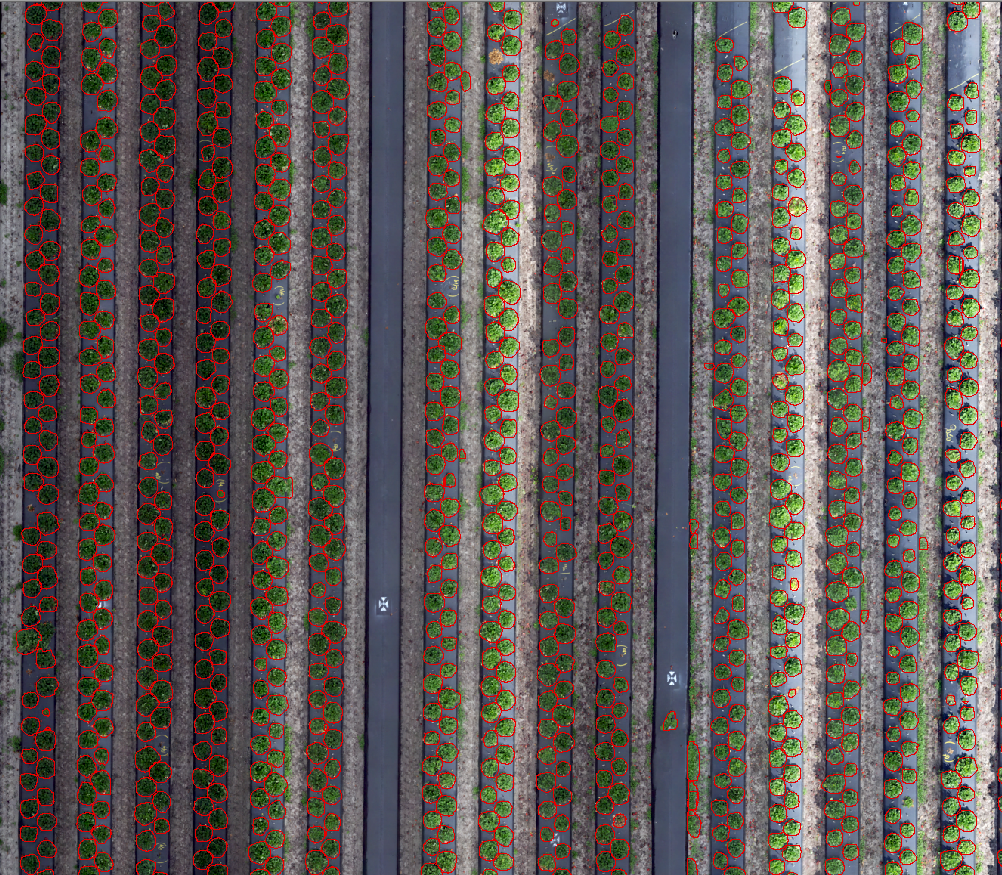


**Figure S4.** Strawberry canopy instance segmentation examples for the image collected at 20210303
